# Pharmacogenomics of *CYP2D6, CYP2C19, CYP2C9,* and Clinical Determinants of Fluoxetine–Norfluoxetine Pharmacokinetics in Real-World Clinical Conditions

**DOI:** 10.3390/pharmaceutics18010041

**Published:** 2025-12-28

**Authors:** Carla González de la Cruz, Levin Thomas, Carmen Mata-Martín, Idian González, Adrián LLerena, Eva M. Peñas-Lledó

**Affiliations:** 1 Personalized Medicine and Mental Health Unit, University Institute for Biosanitary Research of Extremadura (INUBE), 06080 Badajoz, Spain; carla.gonzalezd@externos.salud-juntaex.es (C.G.d.l.C.); levinpharma@gmail.com (L.T.); mariadelcarmen.mata@salud-juntaex.es (C.M.-M.); idian.gonzalez@salud-juntaex.es (I.G.); allerena@unex.es (A.L.); 2 Pharmacogenomics and Personalized Medicine Unit, Badajoz University Hospital, Extremadura Health Service (SES), 06006 Badajoz, Spain; 3 Psychiatry Unit, San Pedro de Alcántara Hospital, Extremadura Health Service (SES), 10002 Cáceres, Spain

**Keywords:** *CYP2D6*, fluoxetine, norfluoxetine, pharmacogenetics, metabolic ratio

## Abstract

**Background:** Fluoxetine, a widely prescribed selective serotonin reuptake inhibitor, exhibits significant interindividual variability in pharmacokinetics, largely attributed to pharmacogenomic factors. **Objectives**: The study aimed to evaluate the impact of pharmacogenetics and clinical determinants on the dose-normalized fluoxetine/norfluoxetine metabolic ratio in patients undergoing fluoxetine therapy in routine clinical settings. **Methods**: Genotypes for *CYP2D6*, *CYP2C9*, and *CYP2C19* genotypes were determined in 47 patients receiving fluoxetine therapy using TaqMan^®^ assays. Steady-state trough plasma concentrations of fluoxetine and norfluoxetine were measured using validated high-performance liquid chromatography methods. Log_10_-transformed dose-normalized fluoxetine/norfluoxetine metabolic ratio (logMR) was compared across *CYP2D6*, *CYP2C9*, and *CYP2C19* genotype-predicted metabolizer groups. Multivariate generalized linear modeling (GLM) was used to evaluate the independent effects of CYP genotypes and clinical covariates on the logMR. **Results**: The logMR differed significantly among the *CYP2D6* genotype-predicted metabolizer groups (*p* < 0.003). *CYP2D6* poor metabolizers exhibited significantly higher logMR than normal metabolizers (*p* < 0.004). The GLM analysis confirmed that *CYP2D6* genotype was the only significant predictor of the logMR independent of all clinical covariates. No significant effects of *CYP2C9*, *CYP2C19* genotypes, or clinical variables on the logMR were observed. **Conclusions**: These findings highlight *CYP2D6* genotype as a key determinant of fluoxetine metabolism during standard treatment. No associations were observed with *CYP2C9* or *CYP2C19* genotypes or clinical factors.

## 1. Introduction

Fluoxetine, one of the most commonly prescribed selective serotonin reuptake inhibitors, remains a cornerstone in the pharmacotherapy for depressive disorders and suicide prevention [1,2]. Despite its long-standing clinical use since its approval by the U.S. Food and Drug Administration (FDA) in 1987, treatment outcomes with fluoxetine show marked interindividual variability, ranging from therapeutic failure to a wide spectrum of dose-limiting adverse drug reactions (ADRs) in psychiatric settings. The variability underscores the persistent challenges in achieving optimal and precise dosing [3,4], which is particularly relevant in the context of suicide risk management. Both the European Medicines Agency and the FDA mandate black box warnings and close monitoring due to the small but clinically significant risk of increased suicidal ideation or behavior (though not necessarily completed suicide) in children, adolescents, and young adults under the age of 25 during the initial weeks of treatment [5]. A key point of ongoing debate is whether this early elevated risk is directly induced by the medication. Regardless of its origin, the heightened vulnerability during the initial phase of treatment underscores the need for rigorous monitoring and continued investigation [6]. Furthermore, antidepressant users who develop ADRs, such as those treated with fluoxetine, are more likely to switch to alternative agents or discontinue treatment entirely [7]. These variable treatment outcomes largely stem from marked interindividual differences in fluoxetine pharmacokinetics across various populations, driven by a complex interplay of pharmacogenomic and clinical factors [8,9]. ADRs also contribute to increased healthcare costs by necessitating additional medical visits, adjunctive treatments, and discontinuation-related expenses, thus offsetting the drug’s low acquisition cost. This collectively highlights the need for pharmacogenomic and pharmacokinetic-informed fluoxetine dosing strategies [10].

Fluoxetine is readily absorbed following oral administration, is extensively plasma protein-bound (>95%), and possesses a large apparent volume of distribution (20 to 42 L/kg) [11]. Furthermore, fluoxetine exhibits a non-linear pharmacokinetic profile, necessitating cautious use in patients with hepatic dysfunction [11]. Fluoxetine is primarily metabolized to its active metabolite, N-desmethylfluoxetine (norfluoxetine), mainly by the CYP2D6 enzyme, and to a lesser extent by CYP2C9 and CYP2C19 [12,13]. Polymorphisms in *CYP2D6*, *CYP2C9*, and *CYP2C19* have been reported to influence the plasma concentrations of fluoxetine and norfluoxetine across diverse populations [8,13,14,15]. Moreover, reduced CYP2D6 activity (e.g., in poor metabolizers, gPMs) may modulate serotonergic neurotransmission. This modulation is hypothesized to contribute to interindividual differences in anxiety-related and social behavioral traits, potentially bearing important functional implications for vulnerability to neuropsychiatric disorders and psychotropic drug responses [16,17]. However, pharmacogenomic variability only partially accounts for the observed heterogeneity in clinical response or ADR susceptibility. Real-world psychiatric practice is characterized by a high prevalence of polypharmacy, age-related physiological changes, and lifestyle factors such as smoking, all of which can affect fluoxetine pharmacokinetics and/or increase the risk of ADRs and suboptimal treatment outcomes [18,19,20,21]. Crucially, the evidence exploring the combined influence of pharmacogenomic and real-world clinical data on fluoxetine exposure variability remains scarce.

The present study, therefore, aimed to elucidate the integrated influence of pharmacogenetics (*CYP2D6*, *CYP2C9*, and *CYP2C19*), demographic, and clinical factors on the dose-normalized fluoxetine/norfluoxetine metabolic ratio in real-world clinical settings. Using a multivariable Generalized Linear Model (GLM) framework, the independent contribution of pharmacogenetics and clinical variables to fluoxetine biotransformation was assessed within a real-world pharmacogenetics implementation setting (MedeA, Extremadura) [22]. Understanding the influence of these multifactorial determinants is essential for designing precision psychiatry initiatives aimed at optimizing fluoxetine efficacy while minimizing adverse effects.

## 2. Materials and Methods

### 2.1. Study Design, Patients, and Ethical Approval

This study included 47 prospectively recruited patients from the MedeA Pharmacogenetics Implementation Strategy (Badajoz, Extremadura, Spain) [22] from August 2021 to December 2024. The inclusion criteria for this study were patients living in Extremadura, who attended the Extremadura Health System, aged 18 years or older, on fluoxetine therapy, and adherent to their prescribed medication regimen. Pregnant women were excluded. All patients provided written informed consent prior to their inclusion in the study. Clinical and demographic variables (including age, sex, smoking status, polypharmacy [≥5 concomitant medications] and hyperpolypharmacy [≥10 concomitant medications]) were evaluated in clinical examination and extracted from electronic medical records. The study was conducted in accordance with the principles of the Declaration of Helsinki and was approved by the Research Ethics Committee for Medicinal Products of Cáceres (CEIm) [No. 052-2021]. The primary endpoint was the relationship between log_10_-transformed dose-normalized fluoxetine/norfluoxetine metabolic ratio (logMR) and *CYP2D6*, *CYP2C9*, and *CYP2C19* genotype-predicted metabolizer phenotypes. The independent contributions of pharmacogenetics, demographic, and clinical covariates to logMR using GLM were the secondary endpoints.

### 2.2. Genotyping

Analysis of cytochrome P450 (CYP450) variant allele (Supplementary Table S1: Supplementary File S1) was performed using commercially available Taqman^®^ gene expression assays (Applied Biosystems, Foster City, CA, USA). PCR plates were analyzed using the ABI 7300 real-time PCR system (Applied Biosystems, Foster City, CA, USA) following the methodology described in previous articles [23]. *CYP2D6* gene duplications and the CYP2D6*5 gene deletion were determined using XL-PCR, as previously described [24]. To predict the enzyme activity, an activity score was assigned to each allele (Supplementary Table S1: Supplementary File S1). Genotype-predicted metabolizer phenotypes were classified following the methods described in a previous study [23].

### 2.3. Pharmacokinetic Analysis

Blood samples were collected in the morning under steady-state conditions, immediately before administration of the subsequent dose. Plasma samples were stored at −20 °C until analysis. A validated commercial kit was used for fluoxetine and norfluoxetine determination from Chromsystems (Chromsystems Instruments & Chemicals GmbH, Munich, Germany) [25]. Plasma samples from patients, calibration standards, and quality control samples were processed as follows: an aliquot of 50 µL of plasma was transferred into a 1.5 mL vial. Subsequently, 25 µL of extraction buffer (**Ref. 92005**) was added, and the mixture was incubated for 2 min. Subsequently, 250 µL of the internal standard solution was added, followed by vortex mixing for 30 s. The samples were immediately centrifuged at 13,000 rpm for 7 min, and finally, 150 µL of the resulting supernatant was transferred into an opaque glass vial and mixed with 150 µL of dilution buffer 1 (**Ref. 92007**). Concomitant medication use was not taken into account for the calculation of metabolic ratios. The samples were analyzed on an Agilent 1200 Series HPLC system (Agilent, Santa Clara, CA, USA) equipped with a binary pump, autosampler, degasser, and column oven. The chromatographic column was a Therapeutic Drug Monitoring (TDM) MasterColumns A (50 mm × 2 mm internal diameter; 3 μm) from Chromsystems MassTox^®^ (**Ref. 92110**) that was kept at a constant temperature of 30 °C. An API2000 triple quadrupole mass spectrometer from AB Sciex (Framingham, MA, USA) equipped with an atmospheric pressure electrospray ionization interface was used for the mass analysis and detection, and operated with Analyst software (version 1.5.1).

### 2.4. Statistical Analysis

Normality of the data was assessed using the Shapiro–Wilk test. Spearman’s rank correlation was used to assess the association between plasma fluoxetine and norfluoxetine concentrations. The logMR was compared across *CYP2D6*, *CYP2C9*, and *CYP2C19* genotype groups using the Kruskal–Wallis test followed by Dunn’s multiple comparisons test. A comparison of logMR between *CYP2D6* poor and non-poor metabolizers was performed using the Mann–Whitney U test.

The association between clinical factors and the logMR was analyzed using a generalized linear model (GLM) independently for each of the *CYP* genes, *CYP2D6*, *CYP2C9*, and *CYP2C19*, with a Gaussian distribution and identity link. The independent covariates included genotype-predicted metabolizer phenotypes, sex, age, smoking status, polypharmacy, hyper-polypharmacy, and fluoxetine total daily normalized dose. One patient was excluded from this analysis due to missing information on smoking status. Model assumptions were assessed by evaluating the normality of the logMR and residuals using the Shapiro–Wilk test, and by testing homoscedasticity across groups using Levene’s test. No major violations were detected, and residual analysis plots confirmed the adequacy of the models. The model was fitted in R (version 4.5.1) using the *glm()* function (stats package). Regression coefficients, standard errors, and *p*-values were derived from the full model to account for all potential confounders. The statistical power of the model was calculated using the *pwr.f2.test()* function from the R pwr package (v 1.3). A *p*-value < 0.05 was considered statistically significant. Statistical tests were performed using GraphPad Prism (version10.6.1) and R (version 4.5.1) software.

## 3. Results

### 3.1. Basic Demographic, Clinical, Pharmacogenetics, and Pharmacokinetic Data

The mean age (SD) of patients was 55.15 ± 14.54 years. The total daily dose of fluoxetine ranged from 20 to 80 mg/day. The demographic, genotype-predicted metabolizer phenotypes, and concomitant CYP inhibitors in the study population have been described in Table 1. The frequencies of the *CYP2D6*, *CYP2C9*, and *CYP2C19* alleles and genotypes are described in Supplementary Tables S2–S7 (Supplementary File S1). The distribution of *CYP2D6*, *CYP2C9*, and *CYP2C19* genotype predicted metabolizer phenotypes across age and gender groups is shown in Supplementary Figure S1 (Supplementary File S1). *CYP2D6* and *CYP2C9* distributions are characterized by a predominance of intermediate gIMs and gNMs across age and gender strata. In contrast, *CYP2C19* showed a broader genotype-predicted metabolizer spectrum, including additional representation of gRMs and gUMs across both age and gender groups. Across *CYP2D6*, *CYP2C9*, and *CYP2C19* genotype-predicted metabolizer phenotypes, median prescribed fluoxetine total daily doses overlapped substantially across age and sex strata, with most patients receiving 20 to 40 mg/day regardless of genotype, age group, or gender (Supplementary Tables S8 and S9, Supplementary File S1). The fluoxetine concentrations ranged from 5.9 to 465.9 ng/mL, whereas the norfluoxetine concentrations ranged from 9 to 1037.1 ng/mL. A total of 27 (57.45%) patients had combined fluoxetine + norfluoxetine (active moiety) concentrations within the recommended therapeutic range of 120–500 ng/mL. Six patients (12.76%) had concentrations below 120 ng/mL, two (4.25%) exceeded 1000 ng/mL, and 12 (25.53%) fell between 500 and 1000 ng/mL. The median (Q1, Q3) fluoxetine/norfluoxetine metabolic ratio of patients aged ≥65 years and <65 years was 0.020 (0.014, 0.034) and 0.026 (0.018, 0.037), respectively. For male and female patients, the median (Q1, Q3) fluoxetine/norfluoxetine metabolic ratios were 0.024 (0.015, 0.042) and 0.025 (0.017, 0.037), respectively.

**Figure 1:**
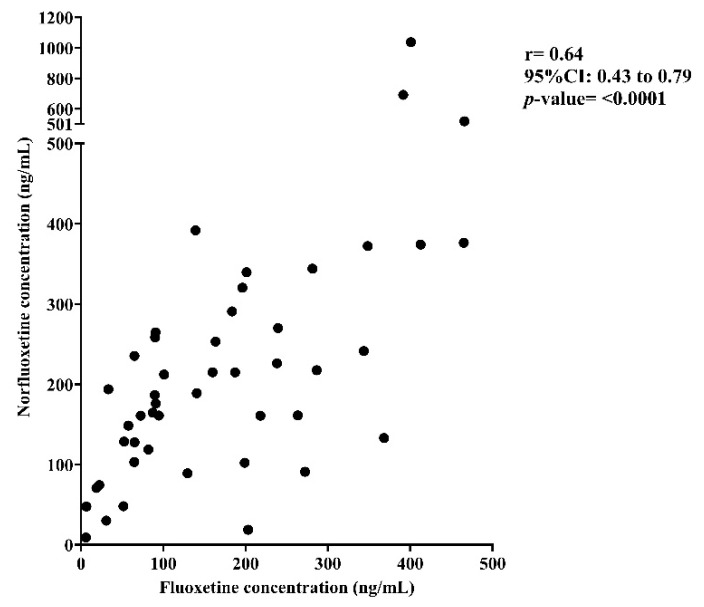
Correlation between plasma fluoxetine and norfluoxetine concentrations.

A significant positive correlation was observed between plasma fluoxetine and norfluoxetine concentrations (Spearman’s r = 0.64, 95%CI: 0.43–0.79, *p* < 0.0001) (Figure 1).

### 3.2. Influence of CYP2D6, CYP2C9, and CYP2C19 Genotypes on Dose-Normalized Fluoxetine/Norfluoxetine Metabolic Ratio

**Figure 2:**
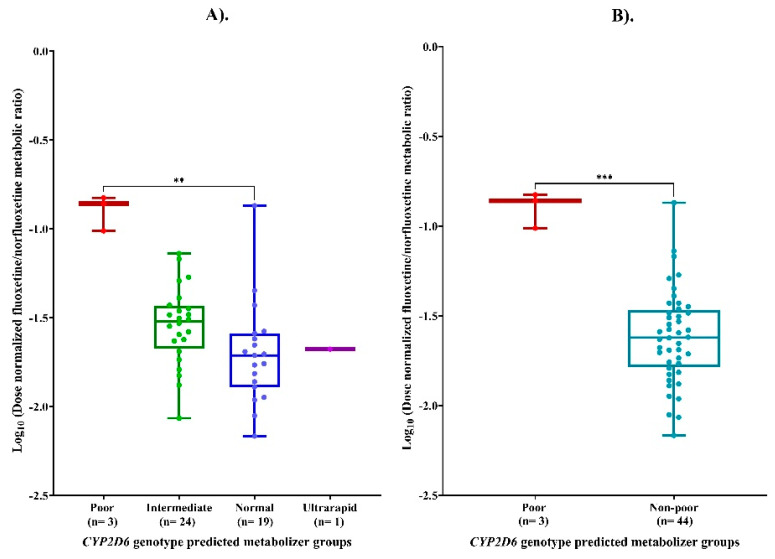
(**A**) Log_10_-transformed dose-normalized fluoxetine/norfluoxetine metabolic ratio (LogMR) across *CYP2D6* genotype groups. (**B**) Comparison of LogMR between poor and non-poor *CYP2D6* metabolizers. Footnotes: ** *p* < 0.01; *** *p* < 0.001.

LogMR differed significantly among the four *CYP2D6* genotypic-predicted metabolizer phenotypes, with gPMs exhibiting higher systemic exposure than gNMs or other metabolizer groups, as shown in Figure 2A (*p* value = 0.003). Post hoc pairwise comparison tests revealed that *CYP2D6* gPMs had significantly higher logMR compared with gNMs (*p* value = 0.004). No other pairwise differences between the *CYP2D6* metabolizer groups reached statistical significance (*p* > 0.05). A statistically significant difference was also observed for logMR between *CYP2D6* gPMs and non-poor metabolizers (*p* = 0.0002), as shown in Figure 2B.

**Figure 3:**
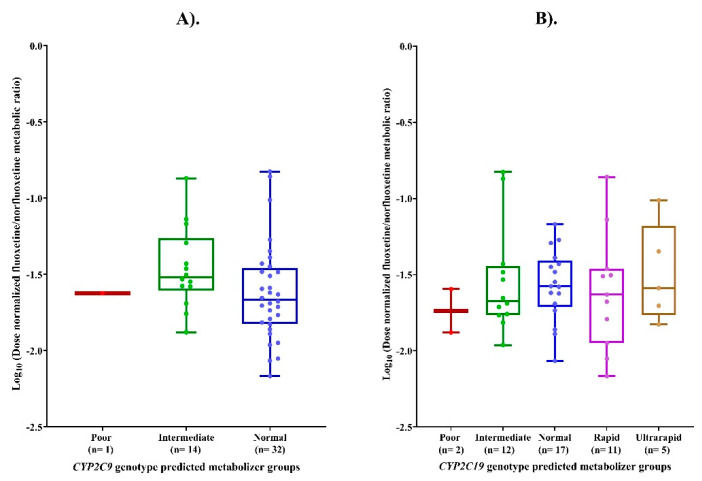
(**A**) LogMR across CYP2C9 genotype-predicted metabolizer phenotypes. (**B**) Comparison of logMR across CYP2C19 genotype-predicted metabolizer phenotypes.

No statistically significant differences were observed in the logMR across *CYP2C9* (*p* = 0.16) and *CYP2C19* (*p* = 0.83) genotype-predicted metabolizer phenotypes. Post hoc pairwise comparison test revealed no significant differences between any individual *CYP2C9/CYP2C19* genotype-predicted metabolizer phenotypes, as shown in Figure 3.

### 3.3. Multivariate Modeling

Normality was confirmed by the Shapiro–Wilk test (*p* = 0.1133), and homogeneity of variances of the logMR across genotype-predicted metabolizer groups was evaluated using Levene’s Test (Supplementary Figure S2, Supplementary File S1). No significant deviations from homoscedasticity were observed (*CYP2D6 p* = 0.447, *CYP2C9 p* = 0.476, *CYP2C19 p* = 0.727). In the multivariate GLM for *CYP2D6* (Table 2), the genotype-predicted metabolizer groups emerged as the main predictor of the logMR. gPMs showed a markedly higher logMR ratio compared to gNMs (β = 0.828, SE = 0.163, *p* < 0.0001). No significant effects were observed for age, sex, smoking status, fluoxetine dose, polypharmacy, or hyper-polypharmacy. The model explained approximately 36% of the variability (R^2^ = 0.369). In contrast, for the *CYP2C9* and *CYP2C19*, none of the evaluated predictors demonstrated a statistically significant association with the logMR. Moreover, the proportion of variance explained by these models was minimal, with R^2^ values of 0.022 for *CYP2C9* and <0.001 for *CYP2C19*. The overall statistical power for the *CYP2D6* GLM model was high (≈0.95).

## 4. Discussion

### 4.1. CYP2D6 and Fluoxetine Metabolism

*CYP2D6* is a highly polymorphic key “pharmacogene”, characterized by single nucleotide polymorphisms, small insertions/deletions, and larger structural variants. It plays a central role in the metabolism of approximately 20% of commonly prescribed drugs across various medical disciplines, including psychiatry, palliative care, oncology, and cardiology [26]. The allele-level distribution of *CYP2D6* in this cohort (e.g., *3, *4, *6-null alleles; *9, *10, *17, *41-decreased-function alleles; *2 and *35-normal-function alleles) explains the stratification of patients into gPM, gIM, gNM, and gUM groups and aligns with the pronounced differences observed in logMR. The 6.38% frequency of *CYP2D6* genotype-predicted gPMs observed in this study is consistent with other reports from European and Spanish populations [27,28]. Most patients were classified as either *CYP2D6* gIMs or gNMs. A similar frequency of gUMs (2.13%), consistent with previous reports from the Spanish population, was observed in the population studied [28]. Patients identified as *CYP2D6* gPMs exhibited significantly higher logMR compared to gIMs, gNMs, and gUMs, as well as the combined group of non-poor metabolizers (gIMs+gNMs+gUMs). This finding suggests reduced CYP2D6-mediated biotransformation of fluoxetine to its active metabolite, norfluoxetine, in gPMs, consistent with reports in other populations [29,30]. Specifically, *CYP2D6* gPMs in this study had a statistically significantly higher logMR than gNMs. Several studies, including systematic reviews and meta-analyses, have reported statistically significant differences in the exposure to various antidepressants, including fluoxetine, between *CYP2D6* gPMs and gNMs [31,32]. GLM analysis confirmed that *CYP2D6* genotype was the only significant predictor of logMR after adjustment for all demographic and clinical covariates.

### 4.2. Role of CYP2C9 and CYP2C19

Additionally, we observed a low frequency of *CYP2C9* gPMs, similar to previously published reports [33,34]. A relatively high proportion of patients in this study were classified as *CYP2C19* gRMs and gUMs, consistent with previous reports from the Spanish and other European populations [35]. Furthermore, one study states that there is no evidence to support the role of the CYP2C9 enzyme in the biotransformation of fluoxetine [12]. Similarly, the reported contribution of CYP2C19 to the metabolic pathway is considered non-significant [36].

These finding supports the role of *CYP2D6* pharmacogenetics variability, rather than *CYP2C9* and *CYP2C19*, as the major determinant of fluoxetine metabolic capacity and interindividual pharmacokinetic variability. A recent report highlighted that *CYP2D6* gPMs and gUMs switched fluoxetine to alternative antidepressant treatment two to three times more often than gNMs, underscoring the need to investigate fluoxetine treatment responses across these genotypes in diverse ethnic populations [7].

### 4.3. Active Moiety Concentration and Clinical Factors

According to “Arbeitsgemeinschaft für Neuropsychopharmakologie und Pharmakopsychiatrie” (AGNP) consensus guidelines, the recommended therapeutic reference range for active moiety (fluoxetine+norfluoxetine) is 120–500 ng/mL, with concentrations exceeding 1000 ng/mL considered potentially toxic [37]. In this study, approximately 13% of patients had active moiety concentrations below the therapeutic range, while about 4% exceeded the upper threshold. Sagahón-Azúa et al. previously reported that ~58% of patients had fluoxetine concentrations within the therapeutic range [29], a value comparable to the 57% observed in our cohort.

A strong positive correlation was identified between plasma fluoxetine and norfluoxetine concentrations in our study, consistent with reports in other populations (r = 0.75, *p* < 0.01) [29]. Previous studies have reported that patients aged ≥65 years have statistically significantly higher fluoxetine concentrations than those who were <65 years [20], and smoking has been correlated with a lower serum concentration of the active moiety [21]. However, in the cohort studied, demographic and prescribing factors, including age, sex, smoking status, polypharmacy, and hyperpolypharmacy, did not significantly influence the logMR. This observation suggests that interindividual variability in fluoxetine metabolism is likely driven predominantly by pharmacogenetics rather than by demographic or prescribing factors. These findings underscore the relevance of genotype-guided pharmacokinetic interpretation as a more reliable approach than demographic-based adjustments. Although no significant differences in logMR were observed when stratified by biological sex, several gender-based differences that extend beyond biological classification, such as self-medication, antidepressant switching, and treatment intensification patterns [38,39,40] were not captured in the study. These behavioral and prescribing dimensions of gender may indirectly influence logMR and warrant dedicated research investigation in future studies.

### 4.4. Genotype-Phenotype Discordance

International consortia such as the Clinical Pharmacogenetics Implementation Consortium and the Dutch Pharmacogenetics Working Group recommend antidepressant prescribing based on *CYP2D6* and *CYP2C19* genotypes [41,42]. However, most of these static guidelines do not always provide an accurate prediction of a patient’s functional drug-metabolizing CYP enzyme activity. In some cases, there is a mismatch between the patient’s genotype-predicted CYP phenotype and their actual CYP phenotype due to phenoconversion [43]. The concomitant use of medications that are inhibitors, substrates, or inducers of CYP enzymes is one of the most common causes of phenoconversion in psychiatric settings [44].

In real-world polypharmacy settings, genotype–phenotype discordance due to drug–drug interaction (DDI)-mediated phenoconversion affects up to 60%, 32.2% and 18.3% of patients with respect to *CYP2D6*, *CYP2C19*, and *CYP2C9*, respectively, thereby reducing predictive accuracy [45]. A retrospective cohort study among 117 patients from a psychiatric outpatient clinic reported that ~10% of the psychiatric patients experienced phenoconversion for either *CYP2C19* or *CYP2D6* [46]. A nearly seven-fold increase in the prevalence of *CYP2D6* phenotypic PMs compared to gPMs were reported, highlighting the potential impact of non-genetic factors such as DDIs, comorbidities, and environmental influences on *CYP2D6* activity [47]. The prevalence of *CYP2D6* PM status, as determined after phenoconversion analysis, is therefore important to consider [47]. In the study, no significant differences in the logMR were observed across polypharmacy and hyperpolypharmacy categories.

### 4.5. Limitations and Future Directions

One of the major limitations of this study was the small sample size, which may have limited the statistical power to identify associations between certain pharmacogenetic and clinical variables with logMR. Although this study reflects real-world prescribing patterns, replication in larger and ethnically diverse cohorts is warranted to confirm the generalizability of these results. Fluoxetine exhibits nonlinear pharmacokinetics [11]; hence, dose-normalized fluoxetine/norfluoxetine metabolic ratios in this study should be interpreted as descriptive and comparative markers of CYP genotype-related metabolic variability rather than as an absolute indicator of dose proportionality.

While we explored the potential impact of polypharmacy and hyperpolypharmacy, we did not assess the influence of DDI-mediated phenoconversion that can substantially alter CYP2D6, CYP2C9, and CYP2C19 metabolic activity, potentially leading to genotype–phenotype discordance. Phenoconversion analysis was not performed in the study because only weak or weak+moderate CYP inhibitors were present, and the number of patients exposed to these inhibitors was limited. The small sample size of CYP inhibitor-exposed patients may have reduced the ability to identify the subtle influence of polypharmacy/hyperpolypharmacy on fluoxetine metabolism. Accounting for time-dependent inhibitory or inductive effects of co-prescribed drugs would further refine the precision of the analysis. Furthermore, the present study did not assess the influence of pharmacogenomics and pharmacokinetics on ADRs and treatment responses (e.g., standardized depression rating scale scores or documented antidepressant switching/discontinuation). Clinically actionable interpretation of the study findings must be approached with caution. Although *CYP2D6* gPMs exhibited significantly higher log MR, the present study cannot define fluoxetine dose-adjustment thresholds. Further population-pharmacokinetic and pharmacokinetic–pharmacodynamic modeling in larger, ethnically diverse cohorts is imperative to determine genotype-guided dose adjustments in clinical practice. TDM may provide additional clinical value when used alongside CYP genotyping, particularly given fluoxetine’s longer elimination half-life and the pharmacological contribution of the active moiety, norfluoxetine. Around 43% of patients in the study had active-moiety concentrations outside the AGNP therapeutic range window, underscoring the relevance of TDM to capture factors not explained by genotype alone. The metabolic ratio may serve as a potential biomarker to identify patients at increased risk of ADRs (gPMs) or suboptimal clinical response (gUMs).

Despite the small sample size and lack of accounting for potential confounders such as DDI-mediated phenoconversion in this study, the observed statistically significant association between the *CYP2D6* genotype and the logMR strongly highlights the predominant role of *CYP2D6* genotype in fluoxetine biotransformation. Nevertheless, the present study reinforces the need for further large, multiethnic cohort investigations to evaluate *CYP2D6*-guided and phenoconversion-informed TDM frameworks for improving the safety and efficacy of fluoxetine treatment. Minimizing the risk of adverse effects is vital, as ~15% of patients experienced fluoxetine-related adverse events assessed by the “Udvalg for Kliniske Undersøgelser” side effect scale [21]. Future studies should aim to assess the influence of fluoxetine pharmacokinetics on the occurrence of ADRs.

## 5. Conclusions

The present study identified *CYP2D6* genotype as the predominant determinant of the logMR. Conversely, *CYP2C9* and *CYP2C19* genotypes, and clinical factors (including age, sex, smoking status, polypharmacy, and hyperpolypharmacy) showed no significant influence on this logMR.

These findings underscore the critical importance of incorporating *CYP2D6* genotyping into routine therapeutic monitoring strategies for fluoxetine. The implementation of pharmacogenomics-based personalized medicine approaches can significantly optimize the efficacy of fluoxetine treatment and minimize the risk of ADRs, particularly in diverse populations and in real-world psychiatric polypharmacy contexts.

## Figures and Tables

**Table 1:** Demographic characteristics, genotype-predicted metabolizer phenotypes, and concomitant CYP inhibitors in the study population.

Sl. No.	Variable	Variable Sub-Groups	N (%)
1	Gender	Male	12 (25.5)
		Female	35 (74.5)
2	Age (years)	≥65	12 (25.5)
		<65	35 (74.5)
3	*CYP2D6* genotype predicted phenotypes	Poor	3 (6.4)
		Intermediate	24 (51.1)
		Normal	19 (40.4)
		Ultrarapid	1 (2.1)
4	*CYP2C9* genotype predicted phenotypes	Poor	1 (2.1)
		Intermediate	14 (29.8)
		Normal	32 (68.1)
5	*CYP2C19* genotype predicted phenotypes	Poor	2 (4.3)
		Intermediate	12 (25.5)
		Normal	17 (36.2)
		Rapid	11 (23.4)
		Ultrarapid	5 (10.6)
6	*CYP2D6* inhibitors	Moderate	1 (2.1)
7	*CYP2C9* inhibitors	Weak	2 (4.3)
8	*CYP2C19* inhibitors	Weak	17 (36.2)
		Weak + Moderate	2 (4.3)

**Table 2:** Multivariate GLM results for each CYP gene (*CYP2D6*, *CYP2C9*, and *CYP2C19*). The table reports the coefficient, Standard deviation (SD), and *p*-value for each independent variable (*p*-values shown in bold indicate statistical significance [*p* < 0.05]). * For categorical variables, the reference categories are Non-smoker (for Smoker and Ex-smoker), Normal metabolizer (for predicted phenotype), Male (for Sex), and No polypharmacy and No hyperpolypharmacy (for Polypharmacy and Hyperpolypharmacy).

Variables	Genes	β	Standard Deviation (SD)	*p*-Value
Intermediate metabolizer *	*CYP2D6*	0.151811	0.076232	0.0543
	*CYP2C9*	0.208125	0.106549	0.0586
	*CYP2C19*	0.0697751	0.1388972	0.619
Poor metabolizer *	*CYP2D6*	0.827953	0.162983	**<0.0001**
	*CYP2C9*	−0.048039	0.320757	0.8818
	*CYP2C19*	−0.2389835	0.2697317	0.382
Rapid metabolizer *	*CYP2C19*	−0.0313340	0.1369672	0.820
Ultrarapid metabolizer *	*CYP2D6*	−0.120587	0.268856	0.6565
	*CYP2C19*	0.0961358	0.1908033	0.618
Age	*CYP2D6*	−0.005103	0.002921	0.0894
	*CYP2C9*	−0.004624	0.003537	0.1993
	*CYP2C19*	−0.0046836	0.0037935	0.225
Sex *	*CYP2D6*	−0.112397	0.097085	0.2548
	*CYP2C9*	−0.090184	0.120917	0.4606
	*CYP2C19*	−0.1249414	0.133031	0.355
Smoker *	*CYP2D6*	0.007603	0.095740	0.9372
	*CYP2C9*	0.153739	0.114106	0.1863
	*CYP2C19*	0.1822903	0.1229596	0.147
Ex–smoker *	*CYP2D6*	−0.090531	0.109405	0.4136
	*CYP2C9*	−0.076657	0.139016	0.5847
	*CYP2C19*	−0.0082266	0.1572017	0.959
Polypharmacy *	*CYP2D6*	0.050273	0.096358	0.6051
	*CYP2C9*	0.145487	0.123533	0.7266
	*CYP2C19*	0.0422056	0.1453921	0.773
Hyperpolypharmacy *	*CYP2D6*	−0.096394	0.098585	0.3349
	*CYP2C9*	−0.043655	0.123883	0.7266
	*CYP2C19*	0.0008628	0.1426763	0.995
Fluoxetine dose	*CYP2D6*	−0.002238	0.004102	0.5888
	*CYP2C9*	−0.005783	0.004959	0.2512
	*CYP2C19*	−0.0075508	0.0053755	0.169

## Data Availability

The raw data supporting the conclusions of this article will be made available by the authors upon request.

## Supplementary Materials

The following supporting information can be downloaded at: https://www.mdpi.com/article/10.3390/pharmaceutics18010041/s1, Table S1: *CYP2D6*, *CYP2C9*, and *CYP2C19* variants and their corresponding Taqman^®^ gene expression assays utilized for Real-Time PCR genotyping, Table S2: *CYP2D6 allelic frequencies*; Table S3: *CYP2D6* genotypes frequencies; Table S4: *CYP2C9 allelic frequencies*; Table S5: *CYP2C9* genotypes frequencies; Table S6: of *CYP2C19 allelic frequencies*; Table S7: *CYP2C19* genotypes frequencies; Table S8: Total daily fluoxetine dose stratified by *CYP2D6*, *CYP2C9*, and *CYP2C19* genotype-predicted metabolizer phenotypes across age (<65 vs. ≥65 years); Table S9: Total daily fluoxetine dose stratified by *CYP2D6*, *CYP2C9*, and *CYP2C19* genotype-predicted metabolizer phenotypes across gender; Figure S1: Distribution of *CYP2D6*, *CYP2C9*, and *CYP2C19* genotype-metabolizer predicted phenotypes across age (A–C) and gender groups (D–F). Figure S2: Residual diagnostics for log_10_-transformed dose-normalized fluoxetine/norfluoxetine metabolic ratio (logMR).

## Abbreviations

The following abbreviations are used in this manuscript:

logMR	Log_10_-transformed dose-normalized fluoxetine/norfluoxetine metabolic ratio
FDA	Food and Drug Administration
ADRs	Adverse drug reactions
GLM	Generalized Linear Model
SNVs	Single-nucleotide variants
gPMs	Poor metabolizers
gUMs	Ultrarapid metabolizers
gIMs	Intermediate metabolizers
gNMs	Normal metabolizers
HPLC	High-performance liquid chromatography
AGNP	Arbeitsgemeinschaft für Neuropsychopharmakologie und Pharmakopsychiatrie
DDI	Drug–drug interaction
