# Supplementary material for: Pharmacogenomics of CYP2D6, CYP2C19, CYP2C9, and Clinical Determinants of Fluoxetine–Norfluoxetine Pharmacokinetics in Real-World Clinical Conditions
Source: Pharmaceutics. 2025 Dec 28;18(1):41. doi: 10.3390/pharmaceutics18010041 (PMC12845259; doi:10.3390/pharmaceutics18010041)

Supplementary File S1

Supplementary Table 1. *CYP2D6, CYP2C9*, and *CYP2C19* variants and their corresponding Taqman® assays utilized for Real-Time PCR genotyping

CYP gene	CYP alleles	rs ID	Nucleotide change	Activity score	Allele functional status	Taqman® assay ID
CYP2D6	**2*	rs16947	2851C>T	1	Normal	C__27102425_10
		rs1135840	4181G>C	1		C__27102414_10
	**3*	rs35742686	2550delA	0	None	C__32407232_50
	**4*	rs3892097	1847G>A	0	None	C__27102431_D0
	**6*	rs5030655	1707T>del	0	None	C__32407243_20
	**9*	rs5030656	2616delAAG	0.25	Decreased	C__32407229_60
	**10*	rs1065852	100C>T	0.25	Decreased	C__11484460_40
	**17*	rs28371706	1022C>T	0.5	Decreased	C___2222771_A0
	**35*	rs769258	31G>A	1	Normal	C_27102444_F0
	**41*	rs28371725	2989G>A	0.25	Decreased	C__34816116_20
CYP2C9	**2*	rs1799853	3608C>T	0.5	Decreased	C_25625805_10
	**3*	rs1057910	42614A>C	0	None	C_27104892_10
	**5*	rs28371686	42619C>G	0.5	Decreased	C_27859817_40
	**6*	rs9332131	10601delA	0	None	C__32287221_20
	**8*	rs7900194	3627G>A	0.5	Decreased	C__25625804_10
CYP2C19	**2*	rs4244285	19154G>A		None	C__25986767_70
	**3*	rs4986893	17948G>A		None	C__27861809_10
	**4*	rs28399504	1A>G		None	C__30634136_10
	**5*	rs56337013	90033C>T		None	C__27861810_10
	**17*	rs12248560	-806C>T		Increased	C____469857_10

Supplementary Table 2. *CYP2D6* allelic frequencies

***CYP2D6* alleles**	**Frequency**
wt	0.31
*2	0.22
*4	0.20
*5	0.01
*6	0.02
*9	0.03
*10	0.01
*17	0.01
*35	0.04
*41	0.11
wtx2	0.01
*2x2	0.02

Supplementary Table 3. Frequencies of *CYP2D6* genotypes

***CYP2D6* genotypes**	**Frequency**
*2/*10	0.02
*2/*2	0.09
*2/*35	0.02
*2/*4	0.09
*2/*41	0.04
*2x2/*4	0.04
*4/*35	0.04
*4/*4	0.04
*4/*41	0.02
*4/*5	0.02
*4/*9	0.02
*41/*41	0.02
*6/*41	0.02
*9/*35	0.02
*9/*41	0.02
wt/*17	0.02
wt/*2	0.11
wt/*4	0.09
wt/*41	0.06
wt/*6	0.02
wt/wt	0.15
wtx2/wt	0.02

Supplementary Table 4. *CYP2C9* allelic frequencies

***CYP2C9* allele**	**Frequency**
*2	0.12
*3	0.06
wt	0.82

Supplementary Table 5. Frequencies of *CYP2C9* genotypes

***CYP2C9* genotype**	**Frequency**
*2/*2	0.02
*3/*3	0.02
wt/*2	0.19
wt/*3	0.09
wt/wt	0.68

Supplementary Table 6. *CYP2C19* allelic frequencies

***CYP2C19* allele**	**Frequency**
wt	0.56
*2	0.15
*4	0.02
*17	0.27

Supplementary Table 7. Frequencies of *CYP2C19* genotypes

***CYP2C19* genotype**	**Frequency**
*17/*17	0.11
*2/*17	0.06
*2/*2	0.02
*2/*4	0.02
*4/*17	0.02
wt/*17	0.23
wt/*2	0.17
wt/wt	0.36

Supplementary Table 8. Total daily fluoxetine dose stratified by *CYP2D6, CYP2C9*, and *CYP2C19* genotype-predicted metabolizer phenotypes across age (<65 vs ≥65 years).

**Genotype-predicted metabolizer phenotypes**	**Age group**	**N**	**Fluoxetine total daily dose (mg/day), median [IQR]**
CYP2D6			
PM	<65	2	20 [20, 20]
PM	≥65	1	20 [NA, NA]
IM	<65	21	20 [20, 40]
IM	≥65	3	40 [20, 40]
NM	<65	11	20 [20, 40]
NM	≥65	8	20 [20, 40]
UM	<65	1	30 [NA, NA]
CYP2C9			
PM	<65	1	20 [NA, NA]
IM	<65	12	20 [20, 20]
IM	≥65	2	20 [20, 20]
NM	<65	22	25 [20, 40]
NM	≥65	10	30 [20, 40]
CYP2C19			
PM	<65	2	20 [20, 20]
IM	<65	7	20 [20, 40]
IM	≥65	5	40 [20, 60]
NM	<65	12	20 [20, 40]
NM	≥65	5	20 [20, 30]
RM	<65	10	25 [20, 40]
RM	≥65	1	40
UM	<65	4	20 [20, 27.5]
UM	≥65	1	20

Footnotes: For strata with n = 1, only the observed dose is reported; quartiles are not defined.PM = Poor metabolizer, IM = Intermediate metabolizer, NM = Normal metabolizer, RM = Rapid metbaolzier, UM = Ultrarapid Metabolizer.

Supplementary Table 9. Total daily fluoxetine dose stratified by *CYP2D6, CYP2C9*, and *CYP2C19* genotype-predicted metabolizer phenotypes across gender.

**Genotype-predicted metabolizer phenotypes**	**Gender**	**N**	**Fluoxetine total daily dose (mg/day), median [IQR]**
CYP2D6			
PM	Male	1	20 [NA, NA]
PM	Female	2	20 [20, 20]
IM	Male	6	20 [20, 30]
IM	Female	18	20 [20, 40]
NM	Male	5	20 [20, 30]
NM	Female	14	20 [20, 40]
UM	Female	1	30 [NA, NA]
CYP2C9			
PM	Female	1	20 [NA, NA]
IM	Male	3	20 [20, 20]
IM	Female	11	20 [20, 20]
NM	Male	9	20 [20, 30]
NM	Female	23	40 [20, 40]
CYP2C19			
PM	Female	2	20 [20, 20]
IM	Male	3	20 [20, 20]
IM	Female	9	40 [20, 60]
NM	Male	3	30 [20, 60]
NM	Female	14	20 [20, 40]
RM	Male	3	20 [20, 20]
RM	Female	8	40 [22.5, 40]
UM	Male	3	20 [20, 30]
UM	Female	2	20 [20, 20]

Footnotes: For strata with n = 1, only the observed dose is reported; quartiles are not defined. PM = Poor metabolizer, IM = Intermediate metabolizer, NM = Normal metabolizer, RM = Rapid metbaolzier, UM = Ultrarapid Metabolizer.

Supplementary Figure 1. Distribution of *CYP2D6, CYP2C9*, and *CYP2C19* genotype-predicted metabolizer phenotypes across age (A-C) and gender groups (D-F).

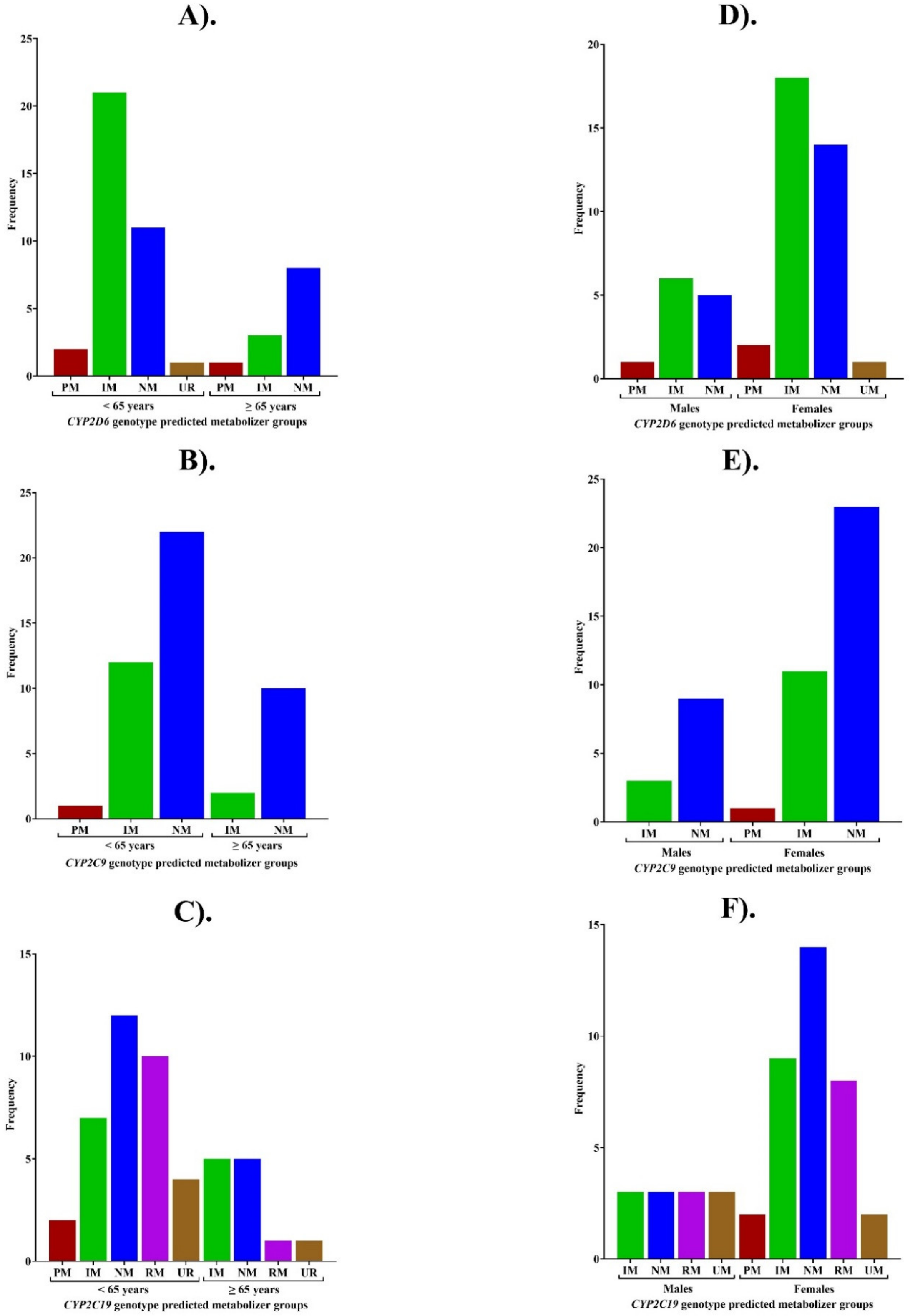

Abbreviations: PM= Poor metabolizer, IM= Intermediate metabolizer, NM= Normal metabolizer, RM= Rapid metabolizer, UR= Ultrarapid metabolizer

Supplementary Figure 2. Residual diagnostics for $\log_{10}$-transformed dose-normalized fluoxetine/norfluoxetine metabolic ratio (logMR).

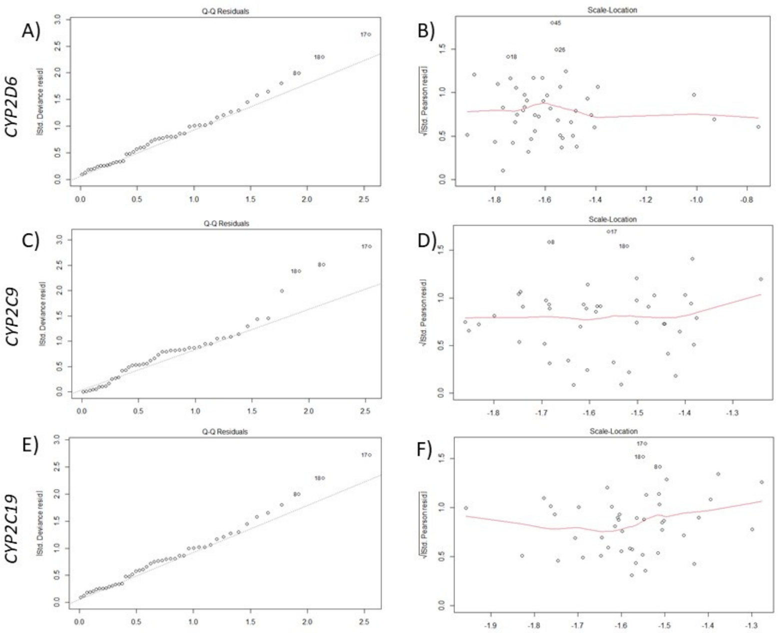

Supplement: Supplementary file 1 [file pharmaceutics-18-00041-s001.zip › pharmaceutics-4029390-supplementary.pdf]
